# A Quarterthiophene-Based Dye as an Efficient Interface Modifier for Hybrid Titanium Dioxide/Poly(3-hexylthiophene)(P3HT) Solar Cells

**DOI:** 10.3390/polym11111752

**Published:** 2019-10-25

**Authors:** Arumugam Pirashanthan, Thanihaichelvan Murugathas, Neil Robertson, Punniamoorthy Ravirajan, Dhayalan Velauthapillai

**Affiliations:** 1 Faculty of Engineering, Western Norway University of Applied Sciences, 5020 Bergen, Norway; pirashanthan.arumugam@gmail.com; 2 Department of Physics, University of Jaffna, Jaffna 40000, Sri Lanka; thanihai@gmail.com; 3 EaStCHEM School of Chemistry, University of Edinburgh, Edinburgh EH93FJ, UK; neil.robertson@ed.ac.uk

**Keywords:** hybrid solar cells, titanium dioxide, poly(3-hexylthiophene), oligothiophene dye, interface modifier, photovoltaic, absorption, quantum efficiency, polymers, efficiency

## Abstract

This work focused on studying the influence of dyes, including a thiophene derivative dye with a cyanoacrylic acid group ((E)-2-cyano-3-(3′,3′′,3′′′-trihexyl-[2,2′:5′,2′′:5′′,2′′′- quaterthiophene]-5-yl) acrylicacid)(4T), on the photovoltaic performance of titanium dioxide (TiO_2_)/poly(3-hexyl thiophene)(P3HT) solar cells. The insertion of dye at the interface improved the efficiency regardless of the dye used. However, 4T dye significantly improved the efficiency by a factor of three when compared to the corresponding control. This improvement is mainly due to an increase in short circuit current density (J_SC_), which is consistent with higher hole-mobility reported in TiO_2_/P3HT nanocomposite with 4T dye. Optical absorption data further revealed that 4T extended the spectral response of the TiO_2_/P3HT nanocomposite, which could also enhance the J_SC_. The reduced dark current upon dye insertion ensured the carrier recombination was controlled at the interface. This, in turn, increased the open circuit voltage. An optimized hybrid TiO_2_/P3HT device with 4T dye as an interface modifier showed an average efficiency of over 2% under-simulated irradiation of 100 mWcm^−2^ (1 sun) with an Air Mass 1.5 filter.

## 1. Introduction

Hybrid nanoporous metal-oxide polymer photovoltaic devices have been intensively studied for more than two decades, as these devices offer potential advantages relative to organic acceptors, such as low cost, facile synthesis via wet chemical processing, control of heterojunction morphology, and the potential for higher physical and chemical stabilities [1]. A metal-oxide nanoparticle (TiO_2_, ZnO) percolation network with thickness in the submicron scale provides a stable and transparent backbone network for free carrier transport in this type of solar cells [2]. However, the power conversion efficiency (PCE) of these hybrid devices is limited due to several reasons, including interfacial carrier recombination [3,4] at the interface, poor mobilities in the metal-oxide polymer nanocomposite, and poor spectral response of the polymer [5,6,7,8]. Typically, the nanoporous metal oxides are the electron acceptors and the π-conjugated polymers are the donors [9,10,11] in hybrid metal-oxide polymer solar cells. The electron transfer from a donor into an acceptor produces a large proportion of charge carrier pairs across the donor/acceptor interface. In that situation, the Coulombic attraction of these bound charge carrier pairs limit the device performance by feeding the recombination effects at the interface [8,12,13,14]. It has been shown that engineering the metal-oxide polymer interface can improve the PCE of hybrid solar cells [12,15,16,17]. Using nanolayers of absorber materials could improve the spectral response and reduce the interfacial recombination [18,19]. Organic dye molecules were also widely investigated as the interface modifier for metal-oxide polymer solar cells. In addition to a number of natural dyes [20,21,22,23,24], N719 and Z907 are two of the most common Ruthenium-based dyes successfully used as an absorber material in highly efficient dye-sensitized solar cells [25,26]. These dyes were also efficiently used as an interface modifier in solid-state hybrid solar cells and were found to improve the spectral response by participating in carrier generation, limiting the recombination [27] at the interface and hence improving both short circuit current density (J_SC_) and the open circuit voltage (V_OC_).

Planells M. et al. reported a series of thiophene derivative dyes with a cyanoacrylic acid group with conjugation length from one to five thiophene units (1T to 5T) as interface modifiers at TiO_2_/P3HT solar cells [16]. These dyes are metal ion-free dyes and have an electron-rich thiophene group. It was found that the dyes improve the V_OC_ due to a dipole moment at the interface [16,28]. Oligothiophenes are discrete, monodisperse molecules, and are distinct from polythiophene, which inherently exists as a distribution of molecular weights. A pure carboxylated oligothiophene can be isolated from any unfunctionalized oligomers via column chromatography and recrystallization [28]. Such organic semiconducting oligothiophenes have been intensively investigated and widely used in organic photovoltaic (OPVs) due to the presence of excellent charge transport properties and tunable optical/electrochemical properties [29]. These tunable electrochemical properties were successfully investigated with variation of thiophene unit and show energy gap reduction when increasing the number of thiophene units from 1T to 5T [16,30].

The 4T dye at the interface was found to increase the hole-mobility in TiO_2_/P3HT polymer nanocomposite by a magnitude of 10 times compared to the corresponding untreated nanocomposite. This is due to passivation of surface traps by the dye, as well as improved packing of the polymer with the nanocrystals through effective inter-chain interactions of 4T with P3HT [8]. The molar extinction coefficient (MEC) is an important parameter in defining the amount of material to be loaded on an electrode for maximum energy conversion, particularly at thin layers of the acceptors. It was also reported that the dyes with higher MEC can improve the stability of dye-based solar cells [31]. Given that the 4T dye can improve the performance of TiO_2_-P3HT solar cells by involvement in photocurrent generation, the amount of dye molecules at the interface needs to be optimized. It was found that the MEC of 4T dye is higher than that of N719 and Z907 dyes. This work enhanced the performance of hybrid TiO_2_/P3HT polymer solar cells by optimizing the device fabrication conditions with dyes and investigates the role of 4T dye at the metal-oxide polymer interface in enhancing the performance of hybrid TiO_2_/P3HT polymer solar cells.

## 2. Materials and Methods 

**Figure 1:**
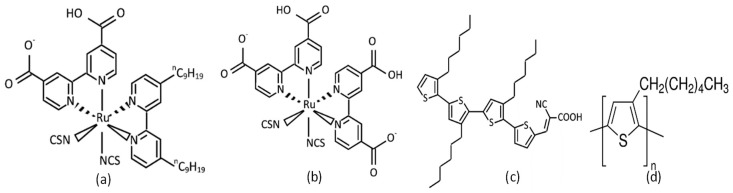
Chemical structure of dyes and polymer used: (**a**). Z907 [8], (**b**). N719 [8], (**c**). 4T [8,16], and (**d**) P3HT.

*Solar Cell Fabrication:* The solar cells were made using indium tin oxide (ITO) coated glass substrates (12 mm × 12 mm, 10 Ω/square). All the chemicals and solvents used in this work were purchased from Sigma Aldrich. The cleaned ITO substrates were first spray-coated with a diluted solution of titanium (iv) isopropoxide and acetylacetone mixture [8] in ethanol at a substrate temperature of 500 °C and baked at the same temperature for 30 min in order to form a ~50 nm-thick dense/blocking TiO_2_ layer. Thereafter, a mesoporous TiO_2_ layer was spin-coated on top of the dense TiO_2_ with the solution (240 mg ml^−1^) of TiO_2_ paste (18NRT) (Dyesol, Queanbeyan, Australia) [30,32,33] dissolved in tetrahydrofuran [8] and allowed to sinter at 450 °C for 30 min [34]. As in previous studies [8,11,35], we used 0.3 mM concentrated dyes Z907 (Mw = 870.10), N719 (Mw = 1188.55), and 4T (Mw = 678.05) by dip-coating for 16 h at 90 °C in order to modify the mesoporous TiO_2_ films. The chemical structures of the dyes and polymer used in this work are shown in Figure 1.

The influence of the concentration of the interface modifier on the device performance was examined with various concentrations of 4T dye. In each situation, the dye solutions were prepared using a 1:1 volume ratio solvent mixture of acetonitrile with tert-butanol [8,30,32]. After dye dipping, the electrodes were washed in 1:1 volume ratio mixture of acetonitrile with tert-butanol to remove excess dye in the nanoporous layer [8]. The dye-modified electrodes were first dip-coated with 2.5 mg ml^−1^ P3HT (Merck KGaA, Germany) and then spin-coated with (25 mg ml^−1^) P3HT solution dissolved in chlorobenzene. Next, 100 nm of Gold top contact was deposited as described in [3,18,19] by thermal evaporation under high vacuum through an Edwards E306 thermal evaporator (Moorfield, Cheshire, UK). Finally, the fabricated solar cell devices were allowed to anneal process with nitrogen medium at 120 °C for 10 min in order to improve the interfacial characteristics [36].

*Optical Characterization:* Absorbance spectra of the dye-coated TiO_2_ films were recorded using a JENWAY 6800 UV/Vis. Spectrophotometer (OSA, UK), which was controlled using Flight Deck software. The thickness of TiO_2_ and P3HT layers were recorded by field emission scanning electron microscopy (FESEM, ZEIS Sigma, UK)

*Electrical Characterization:* The electrical characterization of both polymer and solid-state solar cells were tested, and the current-voltage curves were recorded with a computer-controlled Keithley 2400 source meter unit under the conditions of dark and 100 mW/cm^2^ illuminations of the solar simulator (SCIENCE TECH, Ontario, Canada) with an Air Mass (AM) 1.5 spectral filter. The external quantum efficiency (EQE) measurements were carried out with a Monochromator (Newport, CA, USA) and a calibrated silicon photodiode (Newport, CA, USA).

## 3. Results

**Figure 2:**
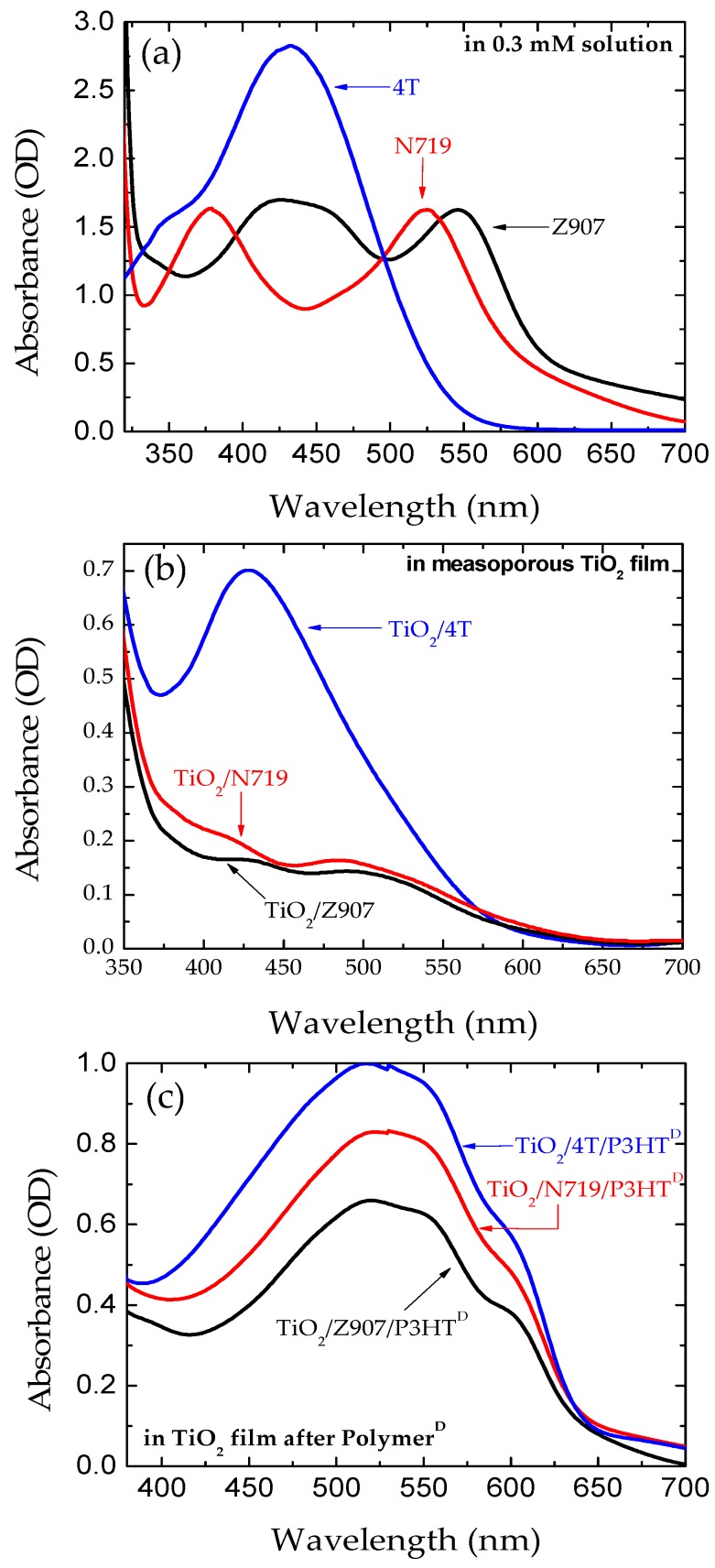
Optical absorption spectra of (**a**) 0.3 mM concentration of dyes dissolved in tert-butanol and acetonitrile solution, (**b**) dye dip-coated nanoporous TiO_2_ electrodes, and (**c**) dye polymer dip-coated nanoporous TiO_2_ electrodes.

Figure 2a–c compares optical absorption spectra of dyes (4T, N719, and Z907) dissolved in tert-butanol and acetonitrile solution with 0.3 mM concentration, dye dip-coated nanoporous TiO_2_ electrodes, and dye polymer dip-coated nanoporous TiO_2_ electrodes. It is clear that the peak MEC of 4T dye is a factor of two higher than the other two standard dyes. P3HT has broader absorption spectrum in the visible region when compared to the metal-complex dyes, and the absorption spectrum of the 4T dye compliments the polymer absorption in the visible region. Figure 2 further shows that the polymer uptake and visible light absorption of the electrode treated with 4T dye was much higher than the electrodes treated with N719 and Z907 dyes. This is probably due to higher MEC of 4T and better compatibility between thiophene based dye and poly(3-hexyl thiophene). The combination of thiophene units of 4T with poly(3-hexyl thiophene) increased the overall thiophene units in the π-conjugated system, which led to the red-shifted and broadened absorbance spectrum under the UV/visible region [37].

**Figure 3:**
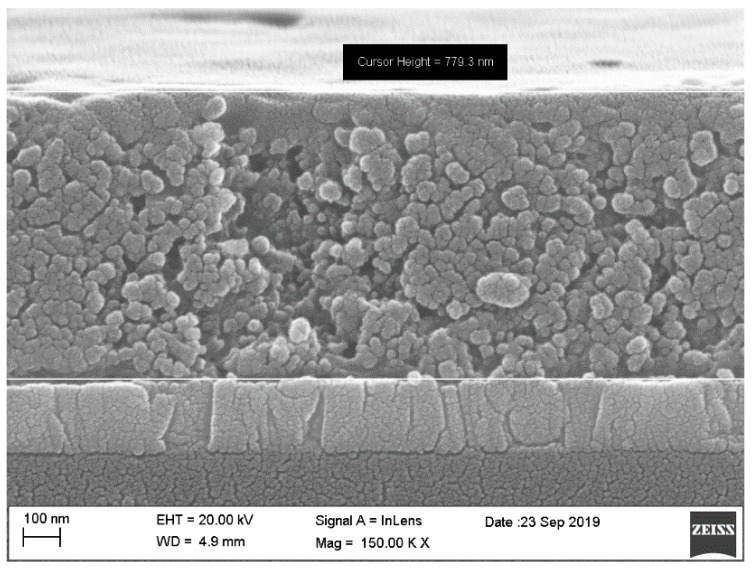
Cross-sectional field emission scanning electron microscopy (FESEM) image for the fabricated TiO_2_/4T/P3HT solar cell.

To confirm the dimensions of the layer, we performed FESEM on the samples. A completed device was cut into two and the cross-section of the device was examined. The cross-sectional FESEM image of the completed device is shown in Figure 3. It clearly shows that thickness of the TiO_2_/4T/P3HT nanocomposite was about 780 nm, in which about 150 nm excess polymer layer could serve as an electron blocking layer (to block direct contact between TiO_2_ nanoparticles and top contact). For FESEM images of each layer, please refer to the Figures SM1–SM4 in the Supplementary Materials.

**Figure 4:**
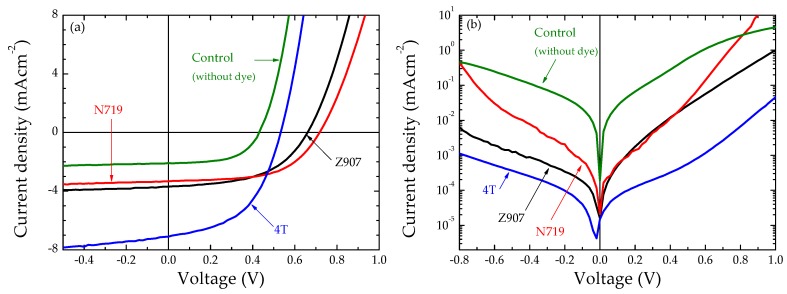
(**a**) J-V characteristics of the fabricated TiO_2_/P3HT and dye-modified TiO_2_/P3HT solar cells under simulated irradiation of 100 mW cm^−2^ (1 sun) with Air Mass 1.5 filter and (**b**) semi-log J-V plot of the solar cells in dark. The complete structure of control device is ITO/TiO_2_/P3HT/Au, and the dye modified cells have the structure ITO/TiO_2_/dye/P3HT/Au. Here, dyes Z907, N719, and 4T were used as the interface modifiers.

**Figure 5:**
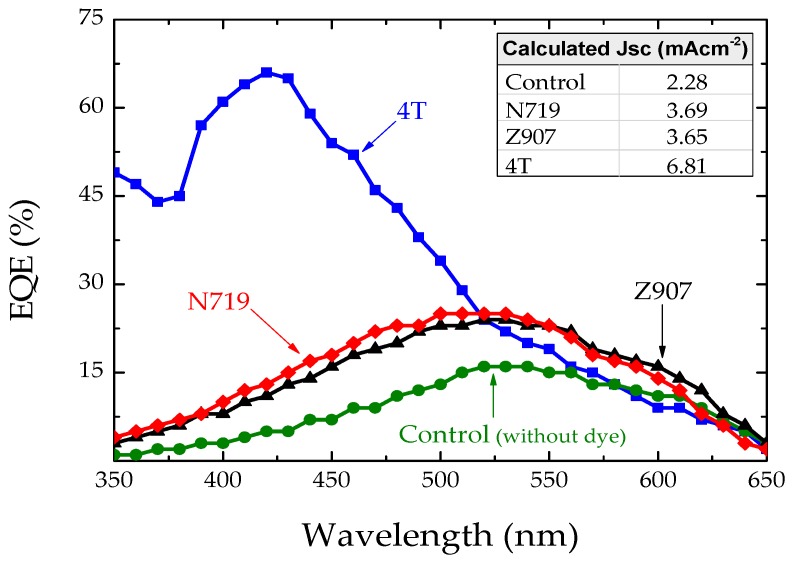
External quantum efficiency (EQE) of ITO/TiO_2_/P3HT/Au (control) and ITO/TiO_2_/dye/P3HT/Au (interface-modified) solar cells. Here, the dyes Z907, N719, and 4T were used as the interface modifiers in TiO_2_/P3HT solar cells. The inset table presents the calculated short circuit current density (J_SC_) from the EQE graph.

Figure 4a and Table 1 clearly show that the insertion of dye molecules at the TiO_2_/P3HT interface increased the short circuit current density (J_SC_), open circuit voltage (V_OC_), and subsequently the PCE. However, the devices with 4T dye-treated electrodes showed a maximum efficiency of about 2%, which was five-fold higher than that of the corresponding control TiO_2_/P3HT devices without any dye treatment. This is mainly due to a four-fold increment in the J_SC_. This can be attributed to increased hole-mobility of P3HT due to the insertion of 4T dye [8] at the interface between the TiO_2_/polymer interface. Figure 4b shows that the dark current was significantly suppressed in dye-treated devices when compared with the corresponding control. This is an indication of reduced back electron transfer [38]. The lowest dark current was observed in 4T dye-treated devices, and was three orders of magnitude lower than that of the corresponding control device. This may suggest that the metal complex dyes have a more beneficial effect in shifting up the TiO_2_ conduction band energy. Then, the external quantum efficiency (EQE) spectra of the dye-treated and untreated TiO_2_/P3HT devices were measured. Figure 5 illustrates the EQE spectra of all devices tested. The conversion efficiency in polymer increased regardless of the dyes used. Figure 5 clearly shows that the influence of dyes N719 and Z907 carrier generations were minimal in the fabricated devices, while influence of 4T dye on carrier generation was dominant, with the peak external quantum efficiency over 60% higher than the peak absorption of 4T dye. This is probably attributed to the improved hole-mobility caused by the 4T dye and better compatibility of the oligothiophene dye with the poly(3-hexyl thiophene) polymer.

**Figure 6:**
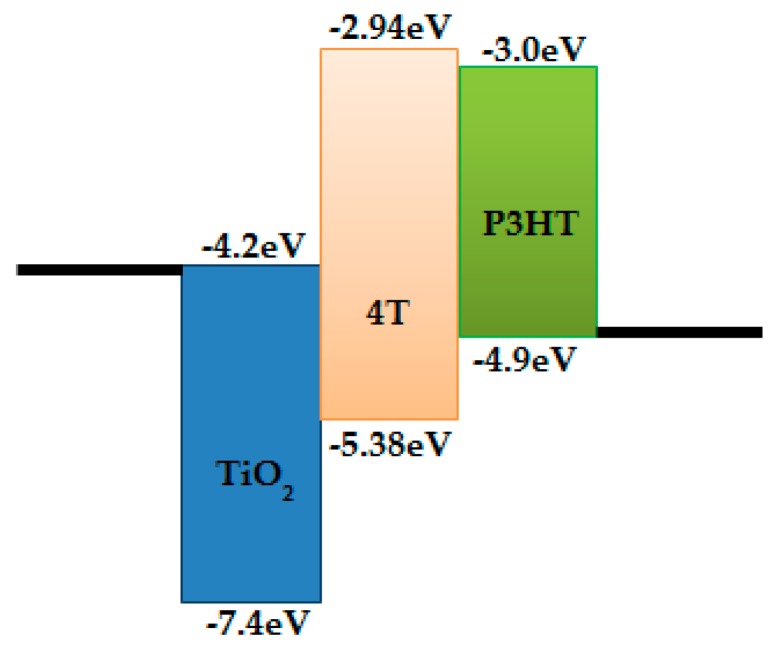
Schematic energy band diagram of the TiO_2_/4T/P3HT solar cell.

Further, Figure 4 shows that dye treatment, especially 4T dye, significantly suppressed the dark current and increased both the V_OC_ and J_SC_ under simulated solar irradiation, relative to the case of the device without dye. This is consistent with the schematic energy band diagram of the TiO_2_/4T/P3HT device shown in the Figure 6, where deep LUMO levels of 4T relative to P3HT were present. The 4T layer was expected to obstruct hole transfer between P3HT and TiO_2_, and thus to localize hole-polarons in the P3HT away from the TiO_2_ surface. Energy levels for TiO_2_ [18], 4T [16], and P3HT [16,18,39] in Figure 6 were directly taken from literature.

**Figure 7:**
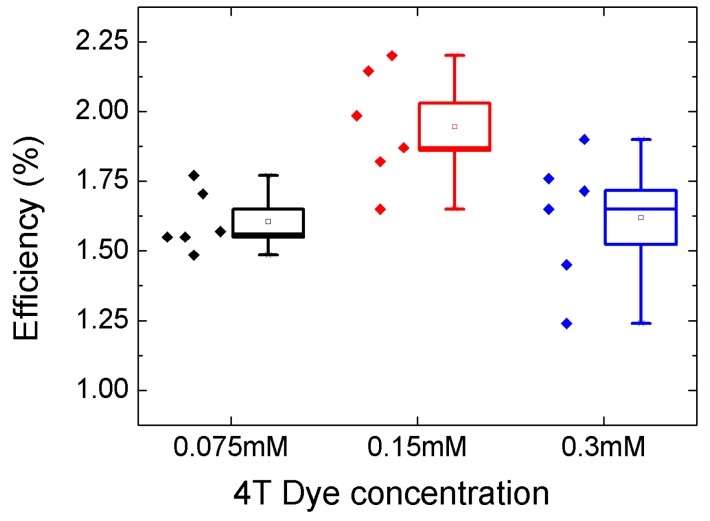
Distribution of power conversion efficiencies of six cells fabricated with three different concentrations (box indicates the standard deviations, whiskers indicate the range, and the small square in the middle of each box indicates the average).

As in Figure 3a, the extinction coefficient of the 4T dye was higher than that of N719 and Z907 dyes. To find the optimum dye concentration for maximum energy conversions, TiO_2_ electrodes with different concentrations of 4T dye were studied. Figure 7 summarizes the variation of photon conversion efficiencies (PCEs) of six cells fabricated with three different concentrations of 4T dye. The average power conversion efficiency was at maximum at 0.15 mM, with a champion efficiency over 2.0%. Table 2 compares the PCEs of solar cells in this work with recently reported TiO_2_/P3HT solar cells with various interface modifiers including dyes. The Table 1 clearly shows that the 4T dye-modified devices showed the best PCE of devices with pristine P3HT and nanoporous TiO_2_ electrodes. It should be noted that our devices have TiO_2_ nanoparticles which did not undergo TiCl_4_ treatment.

## 4. Conclusions

Three different dyes, including a metal-free 4T dye as an interface modifier on TiO_2_/P3HT solar cells, were investigated. It was found that the commercial dyes N719 and Z907 improved the performance of the solar cells by improving the hole-mobility of the polymer and by reducing the back-electron transfer at the interface. Among all the dyes used, the insertion of 4T dye improved the efficiency by five-fold, which was higher when compared to other dyes used. Optimized nanoporous TiO_2_/P3HT solar cells with 4T dye yielded maximum efficiency over 2% under 1 sun illumination with an AM 1.5 filter. This is attributed to a combination of charge carrier generation due to 4T dye, as shown by EQE spectra data, and improved morphology and mobility of the P3HT caused by the 4T.

## Figures and Tables

**Table 1:** Current density vs. voltage measurement data for the control device and other corresponding dye-modified devices.

Condition	$J_{\mathrm{SC}}\left(mAcm^{-2}\right)$	$V_{\mathrm{OC}}\left(V\right)$	FF%	Efficiency %
Without dye (control)	2.09	0.44	44	0.41
N719	3.33	0.65	39	0.86
Z907	3.70	0.71	38	1.01
4T	7.30	0.57	49	2.04

**Table 2:** Photon conversion efficiencies (PCEs) of recently reported TiO_2_/P3HT solar cells with various interface modifiers including dyes.

Device Structure—Different Interface Modifiers	Efficiency %	Year	Reference
TiO_2_/carboxylated oligothiophene/P3HT	0.11	2015	[28]
TiO_2_/BT5 oligomer/P3HT	0.21	2019	[10]
TiCl_4_ treatment/TiO_2_ nanorod/ACA/P3HT	0.28	2015	[40]
TiO_2_/TiCl_4_ treatment/[6,6]-Phenyl C61 butyric acid/P3HT	0.37	2015	[41]
TiO_2_/TiCl_4_ treatment/D131/P3HT	1.53	2015	[41]
TiO_2_/TiCl_4_ treatment/squaraine dye SQ2/P3HT	2.22	2015	[41]
TiO_2_ nanorod/P3HT/PEDOT:PSS	0.43	2012	[15]
TiO_2_ nanorod(650 nm)/D149/P3HT/PEDOT:PSS	1.58	2012	[15]
TiO_2_ nanorod(1.5 μm)/D149/P3HT/PEDOT:PSS	3.12	2012	[15]
TiO_2_ nanorod/Z907/P3HT/PEDOT:PSS	0.94	2012	[15]
TiO_2_ nanowires/Pyridine/P3HT	0.45	2015	[42]
TiO_2_/Z907/P3HT/PEDOT:PSS	0.53	2017	[35]
TiO_2_ nanowires/TiCl_4_ treatment/CdS/P3HT	0.7	2015	[42]
TiO_2_ nanofibers/N719/P3HT	0.90	2010	[43]
TiO_2_/Nitro Benzoic Acid treatment/P3HT/PEDOT:PSS	1.05	2017	[3]
TiO_2_ nanofibers/N719 + PPA/P3HT	1.09	2010	[43]
TiO_2_/Methoxy Benzoic Acid treatment/P3HT/PEDOT:PSS	1.24	2017	[3]
TiO_2_/Al_2_O_3_/N719/P3HT/PEDOT:PSS	1.4	2014	[38]
TiO_2_/TiCl_4_ treatment/4T/doped P3HT	1.54	2014	[16]
TiO_2_/TiCl_4_ treatment/5T/doped P3HT	2.32	2014	[16]
TiO_2_ nanorod/TiCl_4_ treatment/D149/TBP/P3HT/PEDOT:PSS	1.83	2012	[44]
TiO_2_/triphenylamine dye/P3HT	2.01	2016	[45]
TiO_2_/Z907/P3HT	1.01	2019	Current work
TiO_2_/N719/P3HT	0.86	2019	Current work
TiO_2_/4T/P3HT	2.04	2019	Current work

## Supplementary Materials

The following are available online at https://www.mdpi.com/2073-4360/11/11/1752/s1, Figure SM1: Cross sectional FESEM image for fabricated TiO_2_/4T/P3HT solar cell. The thickness of ITO layer was found to be 201.2 nm, Figure SM2: Cross sectional FESEM image for fabricated TiO_2_/4T/P3HT solar cell with individual thickness for each layer, Figure SM3: Cross sectional FESEM image for fabricated TiO_2_/4T/P3HT solar cell. The thickness of P3HT layer was found to be 177.7 nm, Figure SM4: Cross sectional FESEM image for fabricated TiO_2_/4T/P3HT solar cell.
