# Supplementary material for: A Quarterthiophene-Based Dye as an Efficient Interface Modifier for Hybrid Titanium Dioxide/Poly(3-hexylthiophene)(P3HT) Solar Cells
Source: Polymers (Basel). 2019 Oct 25;11(11):1752. doi: 10.3390/polym11111752 (PMC6918415; doi:10.3390/polym11111752)
Supplement: Supplementary file 1 [file polymers-11-01752-s001.pdf]

Supplementary Materials

A quarterthiophene based dye as an efficient interface modifier for Hybrid Titanium dioxide / Poly(3-hexylthiophene)(P3HT) Solar Cells

Arumugam Pirashanthan [1, 2], Thanihaichelvan Murugathas [2], Neil Robertson [3], Punniamoorthy Ravirajan [2,*] and Dhayalan Velauthapillai [1,*]

[1] Faculty of Engineering, Western Norway University of Applied Sciences, 5020 Bergen, Norway; pirashanthan.arumugam@gmail.com , Dhayalan.Velauthapillai@hvl.no

[2] Department of Physics, University of Jaffna, Jaffna 40000, Sri Lanka; pirashanthan.arumugam@gmail.com , thanihai@gmail.com , pravirajan@gmail.com

[3] EaStCHEM School of Chemistry, University of Edinburgh, Edinburgh EH93FJ, United Kingdom; neil.robertson@ed.ac.uk

* Correspondence: pravirajan@gmail.com and Dhayalan.Velauthapillai@hvl.no; Tel.: +94 (0) 71 856 1715 and +47 (0) 92 819 641

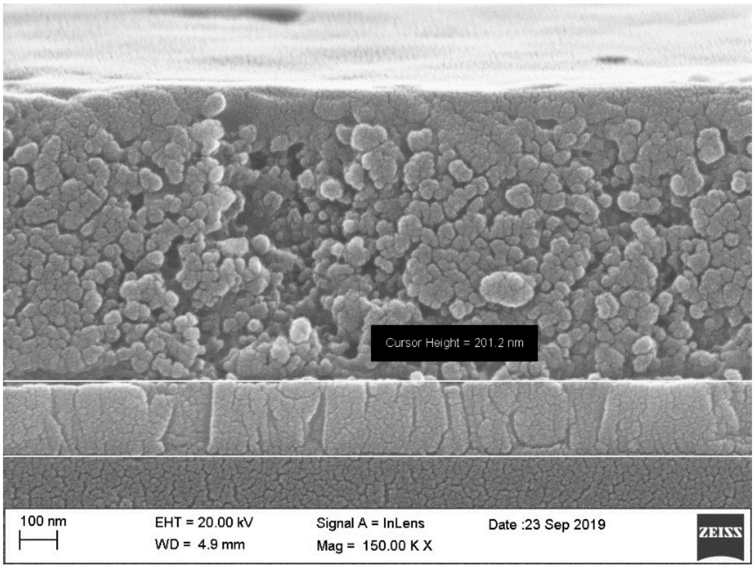

Figure SM1. Cross sectional FESEM image for fabricated TiO_2 / 4T / P3HT solar cell. The thickness of ITO layer was found to be 201.2 nm.

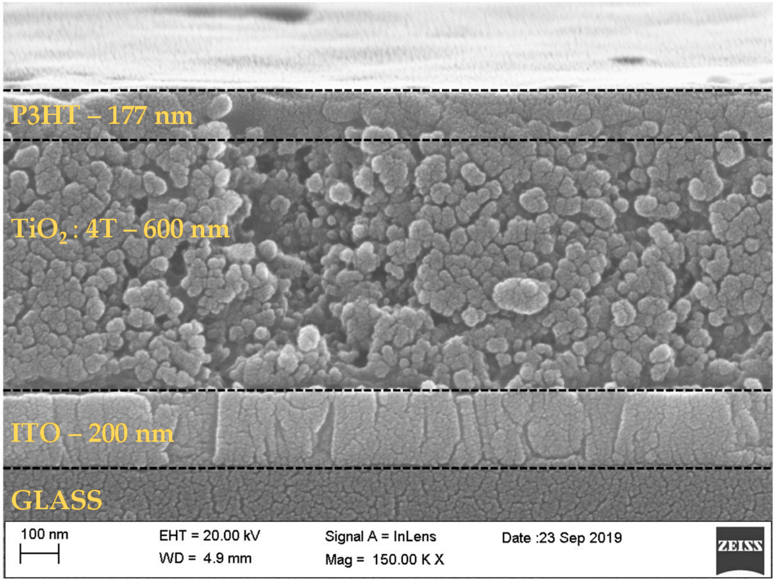

Figure SM2. Cross sectional FESEM image for fabricated TiO_2 / 4T / P3HT solar cell with individual thickness for each layer.

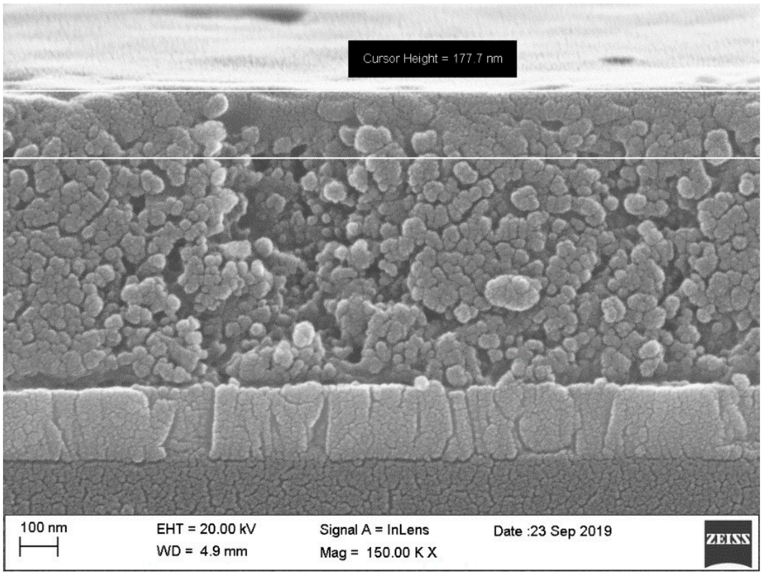

Figure SM3. Cross sectional FESEM image for fabricated TiO_2 / 4T / P3HT solar cell. The thickness of P3HT layer was found to be 177.7 nm.

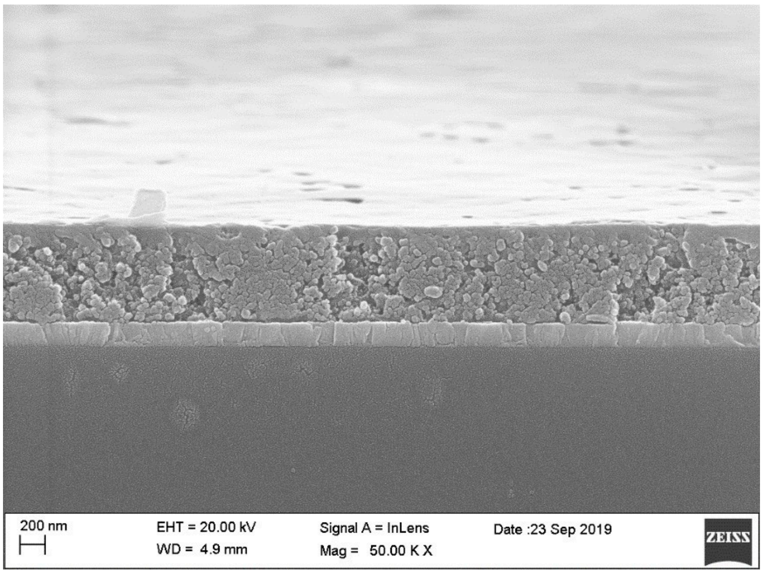

Figure 4. Cross sectional FESEM image for fabricated TiO_2 / 4T / P3HT solar cell.
